# Protective effects of Korean red ginseng against radiation-induced apoptosis in human HaCaT keratinocytes

**DOI:** 10.1093/jrr/rrt109

**Published:** 2013-09-26

**Authors:** Jae Won Chang, Keun Hyung Park, Hye Sook HWANG, Yoo Seob Shin, Young-Taek Oh, Chul-Ho Kim

**Affiliations:** 1 Department of Otolaryngology, School of Medicine, Ajou University, 5 Wonchon-Dong, Yeongtong-Gu, Suwon, 442-749, Korea; 2 Center for Cell Death Regulating Biodrugs, School of Medicine, Ajou University, 5 Wonchon-Dong, Yeongtong-Gu, Suwon, 442-749, Korea; 3 Department of Radiation Oncology, School of Medicine, Ajou University, 5 Wonchon-Dong, Yeongtong-Gu, Suwon, 442-749, Korea

**Keywords:** Korean red ginseng (KRG), head and neck cancer, radiation-induced mucositis, apoptosis, radioprotection

## Abstract

Radiation-induced oral mucositis is a dose-limiting toxic side effect for patients with head and neck cancer. Numerous attempts at improving radiation-induced oral mucositis have not produced a qualified treatment. Ginseng polysaccharide has multiple immunoprotective effects. Our aim was to investigate the effectiveness of Korean red ginseng (KRG) on radiation-induced damage in the human keratinocyte cell line HaCaT and in an *in vivo* zebrafish model. Radiation inhibited HaCaT cell proliferation and migration in a cell viability assay and wound healing assay, respectively. KRG protected against these effects. KRG attenuated the radiation-induced embryotoxicity in the zebrafish model. Irradiation of HaCaT cells caused apoptosis and changes in mitochondrial membrane potential (MMP). KRG inhibited the radiation-induced apoptosis and intracellular generation of reactive oxygen species (ROS), and stabilized the radiation-induced loss of MMP. Western blots revealed KRG-mediated reduced expression of ataxia telangiectasia mutated protein (ATM), p53, c-Jun N-terminal kinase (JNK), p38 and cleaved caspase-3, compared with their significant increase after radiation treatment. The collective results suggest that KRG protects HaCaT cells by blocking ROS generation, inhibiting changes in MMP, and inhibiting the caspase, ATM, p38 and JNK pathways.

## INTRODUCTION

Radiation is the treatment of choice for the majority of head and neck cancers (HNCs). High technology advances such as intensity-modulated radiation therapy and tomotherapy have proved efficacious, although surrounding normal tissues are also affected by irradiation, frequently generating side effects that include dermatitis, xerostomia, and radiation-induced oral mucositis [1]. The lesions are symptomatic and often too severe to maintain treatment and thus become dose-limiting factors. This creates a crucial dilemma for radiation therapy—how to deliver a sufficient radiation dose for oncologic safety while minimizing damage to normal tissue. While strides have been made in managing radiation-induced toxicity, oral mucositis remains a relatively major side effect [2–5].

Oral mucositis is accompanied by altered taste, pain, dry mouth and decreased appetite, and, in severe cases, ulceration, which can decrease nutritional uptake. The consequence can often be a significant impact on the quality of life; and interruption of oral therapy can worsen the prognosis [4]. About 50–90% of cancer patients who undergo radiation therapy experience oral mucositis. Recent studies have shown that the pathogenesis of mucositis is based on loss of epithelial cell renewal, apoptosis, and ulcer formation related to reactive oxygen species (ROS) [6–8]. In our previously published studies, we reported that epicatechin, a minor component of green tea extract, prevents cisplatin-induced ototoxicity and radiation-induced mucositis by attenuating ROS generation and by preventing changes in mitochondrial membrane potential (MMP) [9, 10].

Current oral mucositis therapies are mostly palliative. Some of these include antibiotics, analgesics, general oral hygiene, and the use of various mucosal barrier agents and radioprotective agents, such as amifostine. Despite the clinical significance of oral mucositis, there is no definite approved treatment [11].

Korean red ginseng (KRG, steamed root of *Panax ginseng* CA Meyer) has been an established traditional herbal medicine for millenia. KRG is made by steaming and drying fresh root; the process may result in chemical transformations of molecules including ginsenosides, polysaccharides, peptides, polyacetylenic alcohols, and fatty acids [12]. The spectrum of medicinal effects of KRG include antibacterial [13], antiviral [14], antioxidative [15], antitumor [16], antimutagenic [17], and immune-modulatory activities [18]. Many of these medicinal effects are attributed to the triterpene glycosides known as ginsenosides (saponins) [12].

Since free radicals play an important role in radiation-induced mucosal damage, the underlying radioprotective mechanism of ginseng could be linked, either directly or indirectly, to its antioxidative capability through the scavenging of free radicals. In addition, ginseng's radioprotective potential may also be related to its immunomodulating capabilities [12].

This study assessed the ability of KRG to inhibit radiation-induced oral mucositis in a mucositis cell-line model (human keratinocyte HaCaT cells) as a possible *in vivo* clinical therapy. Associated signaling pathways involving ataxia telangiectasia mutated protein (ATM), p53, p38, c-Jun N-terminal kinase (JNK), and caspase-3 were studied.

## MATERIALS AND METHODS

### Preparation of Korean red ginseng extracts

KRG extracts were provided by Korea Ginseng Corporation (Daejeon, Korea) in a standardized and reproducible process. Briefly, KRG extracts were extracted from red ginseng manufactured from fresh roots of 6-year-old *Panax ginseng* plants whose botanical identity had been verified. Red ginseng was made by steaming fresh ginseng at 90–100°C for 3 h, drying at 50–80°C, extracting seven times with 10 volumes of distilled water at 85°C for 8 h, followed by cooling.

### Cell culture

Human keratinocytes (HaCaT cell line) were obtained from the American Type Culture Collection (ATCC, Manassas, USA). We utilized established HNC cell lines, SCC25 (oral tongue) and SCC1483 (retromolar trigone) purchased from the ATCC. The three cell lines were maintained in high glucose Dulbecco's modified Eagle's medium (DMEM; Gibco, Grand Island, NY, USA) containing 10% fetal bovine serum (FBS; Gibco). The cells were cultured in a humidified incubator at 37°C in an atmosphere containing 5% CO_2_.

### Zebrafish screening model

Mature zebrafish (*Danio rerio*) of the AB wild type strain were purchased from a commercial source. Embryos were obtained by spawning two to four groups of genitors with a male:female ratio of 2:1. We placed the fish in a specific spawning aquarium at 28°C with a mesh bottom to protect the eggs from being eaten. Spawning and fertilization took place within 30 min when the light was switched on in the morning. A single mature female lays 50–200 eggs per day. The embryos were then collected from the aquarium, and 50 embryos per dish were maintained in 100 mm^2^ Petri dishes in specific medium comprised of 1 mM MgSO_4_, 120 µM KH_2_PO_4_, 74 µM Na_2_HPO_4_, 1 mM CaCl_2_, 500 µM KCl, 15 µM NaCl and 500 µM NaHCO_3_ in deionized H_2_O (dH_2_O).

### Cell viability assay

To determine cell viability, cells were seeded in 96-well plates at a density of 3 × 10^3^ cells/well in 0.1 ml complete medium and exposed to various concentrations of KRG (0–100 µM) or radiation plus KRG. After 24 h incubation, the cells were pre-treated with various doses of radiation (2, 4, 8, 15 or 20 Gy), various concentrations of KRG (0, 10, 30, 50 or 100 µg/ml) for 72 h, with or without irradiation (8 Gy), and 40 μl of 3-(4,5-dimethylthiazol-2-yl)-2,5-diphenyl-tetrazolium bromide (MTT) test solution (Sigma-Aldrich, St Louis, MO, USA) was added to each well. The optical density (OD) of each well was measured using an ELISA reader (Bio-Rad, Hercules, CA, USA) at a test wavelength of 596 nm and a reference wavelength of 690 nm. Data were calculated as the percentage of control.

### Treatment effects on zebrafish morphology and survival

Freshly fertilized embryos (6 h postfertilization) were exposed to the radiation (20 Gy) and to different concentrations of the test solution. Four days after exposure, hatching success was recorded. Similarly, survival of embryos was assessed visually at 24-h intervals up to 7 dpf by light microscopy. Mortality was identified by missing heartbeat, coagulation of the embryos, a non-detached tail and failure to develop somites. The morphology was assessed visually using an AXIO vert200 light transmission microscope (Carl Zeiss, Göttingen, Germany) at a magnification of × 60–100. Representative images were recorded using Axiovision software.

### Colony-forming assay

A clonogenic assay was used to elucidate the possible differences in the long-term effects of ginseng on radiation-induced apoptosis of HaCaT cells. Cell suspensions were diluted in DMEM supplemented with 10% FBS, and plated in 6-well plates at 20 cells/cm^2^. After 8 Gy irradiation, cells were treated with 0, 10, 30 or 50 μg/ml KRG for 3 h. After being rinsed with fresh medium, cells were allowed to grow for 8 d to form colonies, which were fixed with 6% glutaraldehyde (Sigma-Aldrich) and stained with 0.5% crystal violet (Sigma-Aldrich). Each plate was photographed and analyzed manually to determine the colony number. Only 200 μm or more colonies were enumerated.

### Wound-healing assay

Investigation of cell migration capability of HaCaT cells was performed using a wound-healing assay, as previously described [19]. Treated and untreated cells were grown to confluent monolayers. Before irradiation, HaCaT cells were treated with each concentration (0, 10, 30 or 50 μg/ml) of KRG. After that, the monolayers were wounded by scratching the surface as uniformly as possible with a 1 ml pipette tip. Then the cells were treated with radiation (8 Gy), the images of the wound area were captured on Day 0 (day of scratching) and Day 2 using an Olympus SC 35 camera connected to an inverted microscope. The migration rate of HaCaT was determined by measuring the percentage closure area. The following formulae were used:

Percentage closure (%) = (migrated cell surface area ÷ total surface area) × 100,

migrated cell surface area = length of cell migration (mm) × 2 × length,

total surface area = 2.4 mm × length.

For each concentration of KRG and each time frame, the experiments were repeated four times.

### Annexin V-fluorescein isothiocynate/propidium iodide staining

Annexin V-fluorescein isothiocynate (FITC)/propidium iodide (PI) staining was processed to quantify the percentage of apoptotic cells at 72 h after irradiation. The cells were stained with an Annexin V-FITC apoptosis detection kit following the manufacturer's protocols (BD Biosciences, Franklin Lakes, NJ, USA). For the assay, the HaCaT cells were cultured overnight in 6-well plates and then irradiated with an 8 Gy dose in the absence or presence (10, 30, 50 μg/ml) of KRG. Following the pretreatments, the cells were washed with PBS three times and resuspended in 1X binding buffer (10 mM HEPES/NaOH at pH 7.4, 140 mM NaCl, and 2.5 mM CaCl_2_). Afterward, the cells were incubated with Annexin V-FITC and PI for 15 min at room temperature and then analyzed by flow cytometry (BD Biosciences).

### Terminal deoxynucleotidyl transferase-mediated dUTP-biotin nick end labeling assay

The terminal deoxynucleotidyl transferase (TdT)-mediated dUTP-biotin nick end labeling (TUNEL) assay method was used to determine apoptosis in the HaCaT cells, using an *in situ* cell detection kit (Roche Molecular Biochemicals, Mannheim, Germany) according to the manufacturer's instructions. HaCaT cells were added to 24-well culture dishes containing growth medium and glass cover slips were placed over them. After cell monolayers achieved 60–70% confluence, the cells were exposed to medium with radiation (8 Gy) in the presence or absence of KRG (10, 30 or 50 μg/ml). Thereafter, the cells were washed with PBS and fixed in 4% paraformaldehyde. The cells were then incubated with 50 μl of TUNEL reaction mixture (TdT and fluorescein-dUTP) at 37°C for 60 min in a humid atmosphere. The cells were stained with Hoechst 33258 (5 μg/ml) for 5 min. The stained cells were analyzed using a fluorescence microscope (Carl Zeiss).

### MMP assessment by JC-1 staining

MMP was determined using flow cytometry with the lipophilic cationic probe 5,5 V,6,6 V-tetrachloro-1,1 V 3,3 V-tetraethylbenzimidazolcarbocyanine iodide (JC-1; Molecular Probes, Eugene, OR, USA). The culture medium was briefly removed from the adherent HaCaT cells and the cells were rinsed with PBS. HaCaT cells with specific treatment were incubated in the dark with JC-1 with DMEM at a final concentration of 10 μM for 30 min at 37°C. The cells were subsequently washed twice with cold PBS and trypsinized. Cell pellets were then resuspended in 500 μl of PBS. The change in MMP was measured by flow cytometry (BD Biosciences) at 72 h after irradiation.

### Measurement of intracellular ROS generation

Intracellular generation of ROS was quantified using 5-(and 6)-carboxyl-2,7-dichlorodihydro fluorescein diacetate (DCFDA; Molecular Probes). This esterified form is cell membrane-permeable and undergoes deacetylation by intracellular esterases. Upon oxidation, DCFDA is converted to highly fluorescent 2,7-dichloro fluorescein (DCF). For the assay, HaCaT cells were cultured overnight in 6-well plates and then treated with 8 Gy of radiation in the presence or absence of KRG for 19 h. The cells were incubated in the dark with 10 μM DCFDA in serum-free medium for 10 min at 33°C. Oxidative burst (hydrogen peroxide, H_2_O_2_) was detected using flow cytometry with excitation and emission settings of 488 and 530 nm, respectively.

### Western blot analysis

Total proteins were extracted using RIPA (Sigma-Aldrich). Protein concentrations were measured using the DC protein Assay (Bio-Rad, Hercules, CA, USA). The proteins were separated by electrophoresis on 12% and 10% sodium dodecyl sulfate polyacrylamide gels. An equal amount of protein (10 μg) was loaded in each lane. After electrophoresis, the proteins were transferred onto polyvinylidene difluoride (PVDF) membranes. Each membrane was blocked with Tris-Buffered Saline Tween-20 (TBST) containing 5% skim milk for 1 h, followed by overnight incubation at 4°C with primary antibody. After washing the membrane extensively, incubation with horseradish peroxidase-conjugated secondary antibody (1:1000, Cell Signaling Technology, Danvers, MA, USA) was performed for 1 h at room temperature. Protein bands on the blots were visualized by ECL Plus Western Blot detection reagents (Amersham Pharmacia Biotech, Piscataway, NJ, USA).

### Statistical analyses

The Student *t*-test and one-way ANOVA were used for the statistical analyses of the data using SPSS 18.0 statistical software (SPSS, Chicago, IL, USA). Parameters of the data from four independent experiments are expressed as the mean ± S.D. *P* < 0.05 was considered statistically significant (*P < 0.05; **P < 0.01; ***P < 0.001).

## RESULTS

### Korean red ginseng increases HaCaT cell viability after irradiation

**Figure 1:**
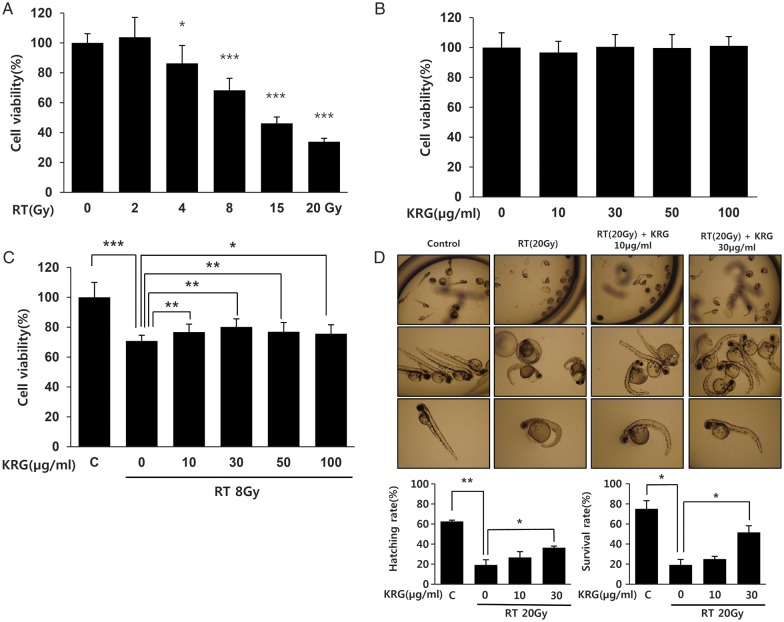
Effect of KRG on viability of the HaCaT cell lines after treatment with radiation. HaCaT cells were exposed to various doses of radiation (0–20 Gy) or various concentrations of KRG (0–100 µg/ml) or radiation plus KRG. At 72 h after radiation exposure, cell viability was measured by an MTT assay. (**A**) Radiation decreased cell viability dose-dependently. (**B**) KRG did not have any influence on the viability of HaCaT cells. (**C**) Cells were pre-treated with an 8 Gy single dose of radiation followed by treatment with 10, 30, 50 or 100 µg/ml KRG for 72 h. KRG significantly protected HaCaT cells from radiation-induced cytotoxicity in a dose-dependent manner. (**D**) Embryos were exposed to radiation (20 Gy) at 6 h postfertilization in the presence or absence of an increasing concentration of KRG (10 and 30 µg/ml). Irradiation induced malformation and decreased hatching/survival rates of zebrafish embryos, but KRG significantly attenuated these embryotoxic effects of the radiation. The data represent the mean ± SD of four independent experiments. *P < 0.05, **P < 0.01, ***P < 0.001.

Radiation decreased the viability of the HaCaT cells in a dose-dependent manner (Fig. 1A). KRG did not show any significant toxic effect on the HaCaT cells (Fig. 1B). We next investigated the effect of various concentrations of KRG (0, 10, 30, 50 and 100 μg/ml) on the radiation-treated cells and discovered that KRG significantly protected the HaCaT cells from radiation-induced cytotoxicity. However, these protective effects did not increase with increasing concentrations of KRG (Fig. 1C). To determine KRG-associated embryotoxic effects in an *in vivo* system, we exposed zebrafish embryos at 6 h post fertilization to 20 Gy of radiation plus various concentrations of KRG (0, 10 and 30 μg/ml) and investigated the effects of this treatment on their morphologic appearance and survival up to 4 dpf of development. Radiation decreased both hatching and survival rates of the zebrafish embryos. Both hatching and survival rate were improved when the zebrafish embryos were treated with KRG, with 30 μg/ml of KRG having a statistically significant protective effect (*P* < 0.05). Also, fewer morphologic abnormalities induced by irradiation, such as microcephaly, decreased lateral line (neuromast) and inhibition of yolk sac resorption, were found in the KRG-treated zebrafish embryos in a KRG dose-dependent manner (Fig. 1D).

### Korean red ginseng does not decrease the therapeutic effect of radiation against cancer cells

**Figure 2:**
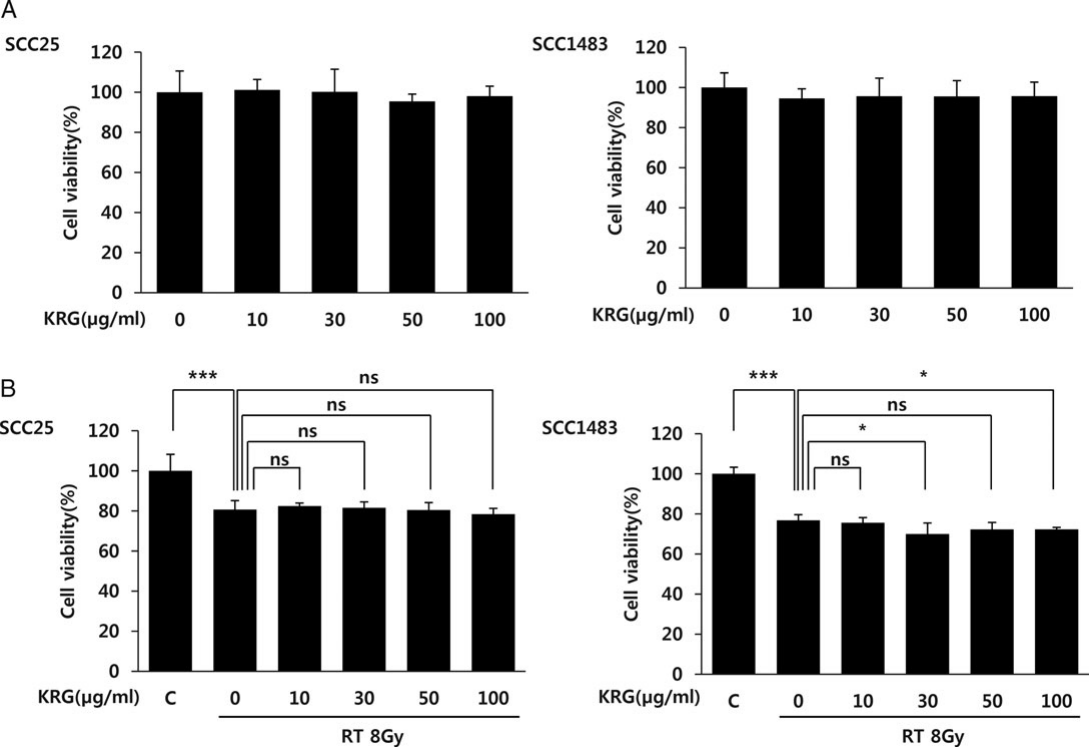
Effect of KRG on the viability of the HNC cell lines after treatment with radiation. (**A**) KRG did not have any influence on the viability of two HNC cell lines (SCC25 and SCC1483). (**B**) SCC25 and SCC1483 cell lines were pre-treated with an 8 Gy single dose of radiation followed by treatment with 10, 30, 50 or 100 µg/ml KRG for 72 h. Then, cell viability was measured via an MTT assay. Radiation decreased cell viability significantly in both cell lines. KRG treatment did not decrease the anticancer effect of irradiation in either cell line. Moreover, 30 and 100 µg/ml KRG decreased the viability of SCC1483 cells compared with radiation treatment alone. The data represent the mean ± SD of four independent experiments. *P < 0.05, **P < 0.01, ***P < 0.001.

If KRG decreases the effect of anti-cancer treatment, it might not be applied in the clinical setting in spite of its protective property with regard to radiation-induced mucosal damage. Therefore we investigated the effect of KRG on the therapeutic effect of radiation treatment of cancer cells derived from HNC (SCC25 and SCC1483). As for the normal keratinocyte HaCaT cells (Fig. 1B), KRG treatment alone did not result in a decrease in cancer cell viability (Fig 2A). In Fig. 2B we see that radiation significantly decreased the viability of the two squamous cancer cell lines (*P*<0.001). However, KRG did not show any significant effect on the viability of the SCC25 cell line. Furthermore, in the SCC1483 cell line, 30 and 100 µg/ml of KRG increased the therapeutic effect of radiation significantly (*P* < 0.05) (Fig. 2B). The results of this experiment show that KRG does not decrease the effect of radiation treatment on the cancer cells, even though it is providing the normal mucosal cells with a level of protection against radiation.

### Korean red ginseng increases clonogenic survival after radiation

**Figure 3:**
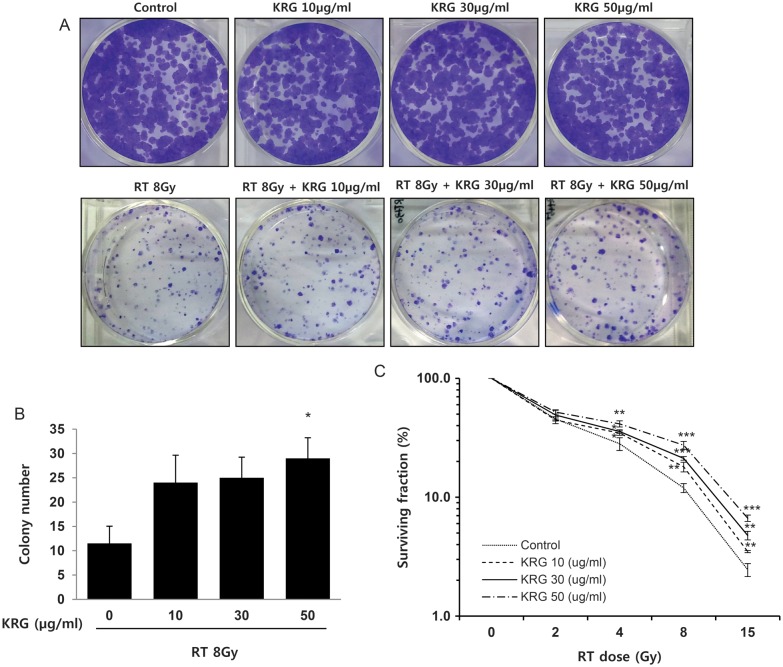
Long-term protective effects of KRG on the ability to form colonies on soft agar. HaCaT cells were treated with KRG (0, 10, 30 or 50 µg/ml) for 3 h. After being rinsed, cells were incubated for 8 days to form colonies and stained with 4% crystal violet. More than 200 µm of the colonies were counted. (**A**) Representative dishes by colony-forming assay. (**B**) Irradiation significantly decreased clonogenic survival of human keratinocytes. KRG increased clonogenic survival after radiation treatment. (**C**) Survival curves of HaCaT cells after irradiation. KRG showed a significant long-term protective effect on the cells, in a dose-dependent manner. The data represent the mean ± SD of four independent experiments. *P < 0.05, **P < 0.01, ***P < 0.001.

A clonogenic assay was used to elucidate the possible differences in long-term effects of KRG on HaCaT human keratinocyte cells. Colony formation in soft agar was obviously decreased by irradiation and was increased in the KRG pretreatment group in a concentration-dependent manner compared with the irradiation-alone group (Fig. 3A); only the 50 μg/ml KRG dose was statistically significant (Fig. 3B). As shown in Fig. 3C, the survival curve of the cells was shifted more and more to the right side with increasing concentration of KRG (0, 10, 30 and 50 μg/ml), indicating a long-term protective effect of KRG treatment.

### KRG restores the migratory ability of HaCaT cells, which is decreased after irradiation

**Figure 4:**
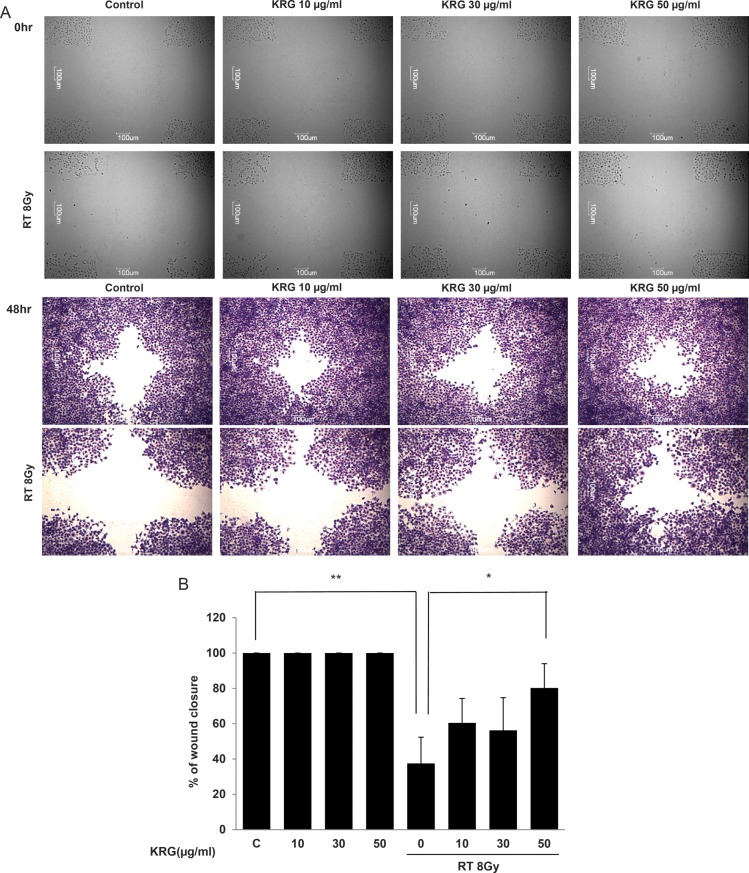
KRG restored cell proliferation and migration, which had been decreased by irradiation. Investigation of cell migration capability after irradiation treatment was performed. (**A**) Confluent monolayers of HaCaT cells were wounded by scratching the surface as uniformly as possible with a 1 ml pipette tip. After treatment with/without an 8 Gy single dose of irradiation, the cells were cultivated for another 48 h. Wound-healing results were visualized with hematoxylin and eosin staining. (**B**) Percentage closure of wound areas was measured. KRG treatment significantly restored cell proliferation and migration at a dose of 50 µg/ml. The data represent the mean ± SD of four independent experiments. *P < 0.05, **P < 0.01.

A wound-healing assay was done to determine the migratory capability of HaCaT cells. Wound healing was monitored at 0 and 48 h (Fig. 4A). All panels treated with radiation showed reduced wound healing capability. Reductions ranged from 50% to 65% 48 h after scratching. However, KRG restored the proliferation and migratory ability in a dose-dependent manner. Proliferation and migration were restored by up to ∼ 80% at the 50 μg/ml concentration (Fig. 4B).

### Korean red ginseng inhibits radiation-induced apoptosis in HaCaT cells

**Figure 5:**
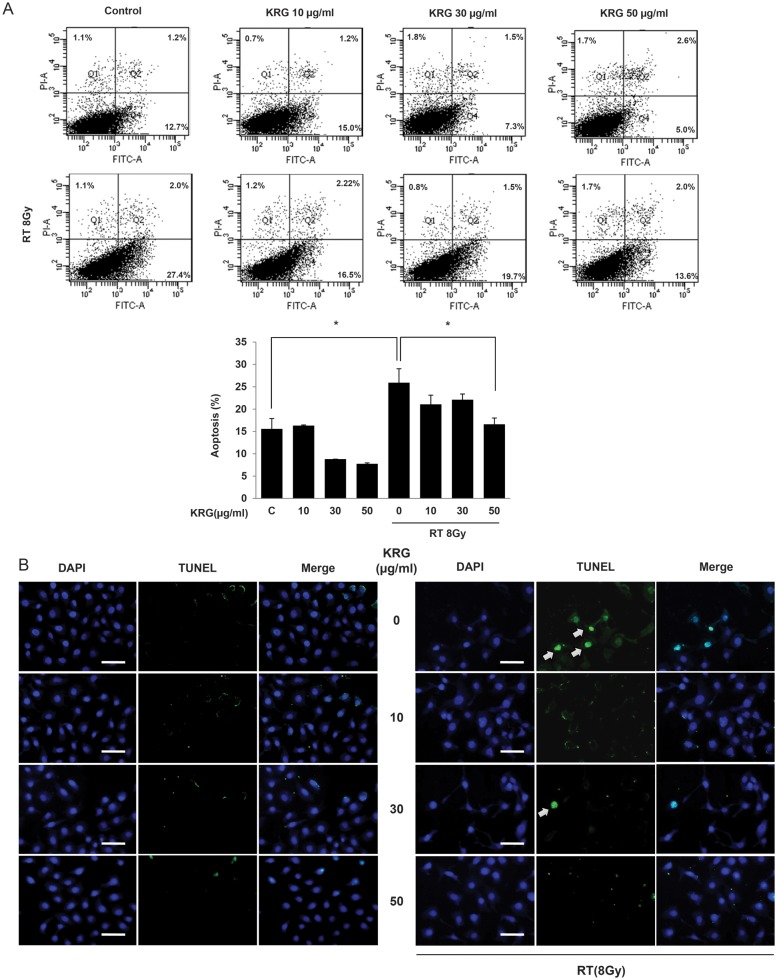
Effect of KRG on radiation-induced apoptosis in HaCaT cells. (**A**) In order to analyze quantitatively the effects of KRG on radiation-induced apoptosis, we used the Annexin V-FITC and PI assay. The percentage of apoptotic cells of those treated with radiation in the absence or presence of KRG were indicated above (upper). The percentage of apoptosis in each fraction was expressed as a graph (lower). KRG significantly decreased apoptosis at a dose of 50 µg/ml. The data represent the mean ± SD of four independent experiments. *P < 0.05. (**B**) Apoptosis in HaCaT cells was identified by the TUNEL method using an *in situ* cell detection kit. After cell monolayers reached 60–70% confluence, the cells were exposed to a medium with radiation (8 Gy) in the presence of 0, 10, 30 or 50 µg/ml of KRG. The cells were then incubated with 50 µl of TUNEL reaction mixture (TdT and fluorescein-dUTP) and stained with Hoechst 33258 (5 µg/ml). The stained cells (arrow) were observed by fluorescence microscopy. The TUNEL assay confirmed that radiation induced TUNEL-positive cells (arrow) and KRG decreased TUNEL-positive cells. Scale bar = 50 µm.

Flow cytometry was used to quantify and verify the number of apoptotic cells induced by irradiation of the HaCaT cells. Annexin V-FITC and PI staining were used to analyze the percentage of apoptotic cells of those treated with radiation in the absence or presence of KRG (Fig. 5A). Radiation treatment increased the Annexin V-FITC/PI-positive fraction of cells significantly (*P* < 0.05), and radiation plus 50 μg/ml KRG treatment significantly decreased (mean 13.6%) the number of apoptotic cells compared with the cells treated with radiation only (mean 27.4%) (*P*<0.05). When HaCaT cells were treated with KRG alone, no significant change in Annexin V-FITC/PI-positive cells compared with the control was observed. These results suggest that radiation promotes apoptotic cell death and that radiation-induced apoptosis can be inhibited by KRG pre-treatment.

TUNEL staining was carried out to determine if radiation-induced apoptosis of HaCaT cells occurred by apoptosis and if this could be inhibited by KRG treatment. Irradiation increased the number of TUNEL-positive cells and KRG treatment decreased the number of TUNEL-positive cells (Fig. 5B). Moreover, as was evident in the RT + KRG panel, KRG decreased DNA fragmentation, perinuclear apoptotic bodies, and nuclear condensation induced after irradiation. These results indicate that KRG inhibited radiation-induced apoptotic cell death.

### KRG inhibits intracellular ROS generation by radiation

**Figure 6:**
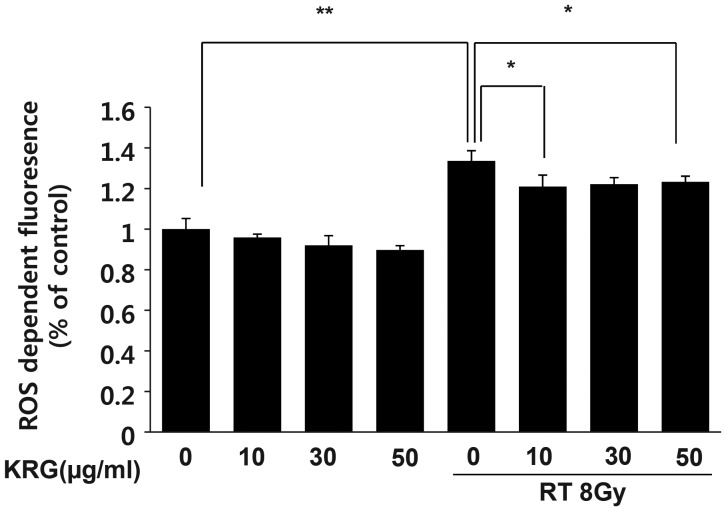
Effect of KRG on radiation-induced intracellular ROS generation in HaCaT cells. For measurement of ROS generation, HaCaT cells were cultured overnight in 6-well plates and then treated with the KRG at 0, 10, 30 or 50 µg/ml in the presence or absence of irradiation (8 Gy). The level of intracellular ROS was measured by flow cytometry using the peroxide-sensitive fluorescent probe, DCFDA. The results (mean ± SD) were calculated as a percentage of the control group (not exposed to radiation). Radiation obviously increased the generation of intracellular ROS. KRG significantly inhibited the intracellular ROS generation induced by irradiation. The data represent the mean ± SD of four independent experiments. *P < 0.05, **P < 0.01.

Next, we analyzed the effect of radiation on intracellular ROS generation. HaCaT cells were treated with 8 Gy of radiation, and the level of intracellular ROS was detected using flow cytometry with the peroxide-sensitive fluorescent probe, DCFDA. Irradiation increased the formation of intracellular ROS significantly, and KRG inhibited radiation-induced ROS generation significantly, regardless of the concentration (Fig. 6).

### Korean red ginseng protects mitochondria by stabilizing MMP

**Figure 7:**
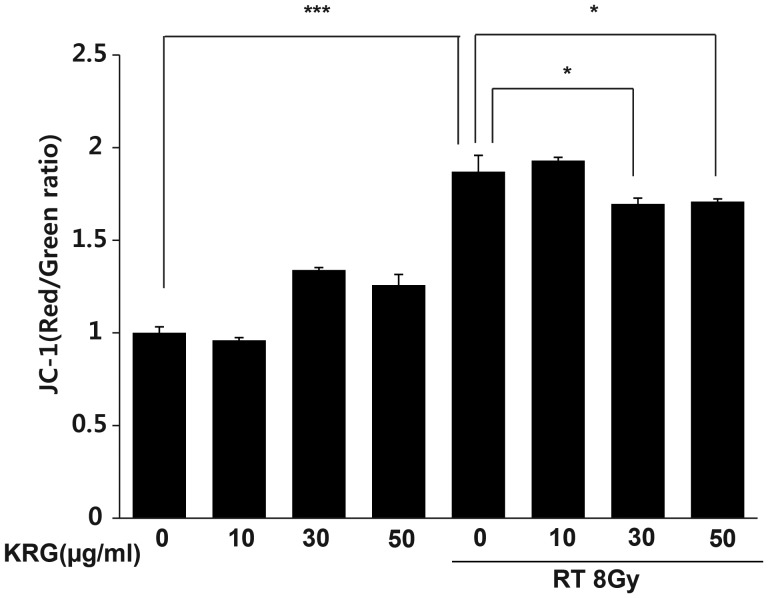
Effect of KRG on mitochondrial membrane potential in HaCaT cells. Cells were treated with or without radiation in the absence or presence of KRG, stained with JC-1, and measured using FACScan. KRG treatment alone did not affect the MMP of HaCaT cells. Stabilization of MMP in the radiation-treated HaCaT cells by KRG was noted (*P* < 0.05). The data represent the mean ± SD of four independent experiments. *P < 0.05, ***P < 0.001.

MMP can be used as an indicator of mitochondrial damage. Therefore, we checked the effect of radiation on MMP in the HaCaT cells to determine whether the loss of MMP could play an important role in radiation-induced apoptosis. MMP is an important parameter of mitochondrial function and is used as an indicator of cell health. JC-1 is a lipophilic, cationic dye that can selectively enter into mitochondria and reversibly change color from green to red as the membrane potential increases. In healthy cells with high MMP, JC-1 spontaneously forms complexes known as J-aggregates with intense red fluorescence. On the other hand, in apoptotic or unhealthy cells with low MMP, JC-1 remains in the monomeric form, which shows only green fluorescence. Control and KRG-only treated cells maintained a high MMP, indicated by the red fluorescence of the JC-1 dye. However, irradiation clearly increased the green color, indicating a loss of MMP (*P*<0.001). KRG partially restored red fluorescence (Fig. 7). Therefore, KRG inhibited radiation-induced changes in the MMP.

### Korean red ginseng rescues HaCaT cells by inhibiting the caspase-3 pathway and the activation of ATM and p53

**Figure 8:**
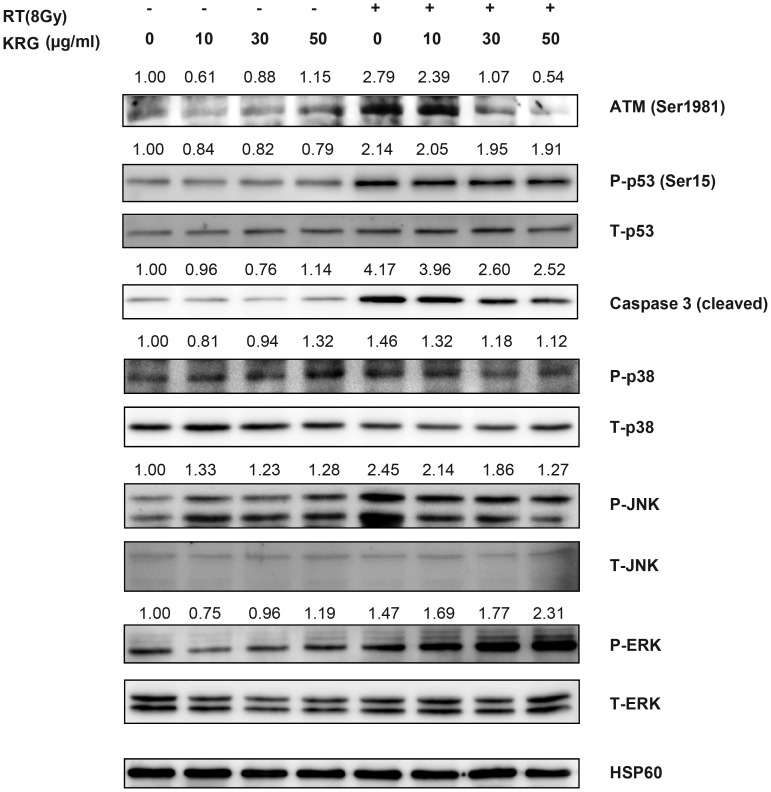
Effect of KRG on radiation-induced apoptosis, ATM-p53, or MAPK expression in HaCaT cells. The cells were incubated for 72 h after treatment with/without radiation and KRG. Cell lysates were collected, gel electrophoresed, and the amounts of ATM, p53, ERK, JNK, p38 and cleaved caspase-3 were measured by Western blot analysis.

To investigate the signals related to radiation-induced cell death and the mechanism of the protective effect of KRG in HaCaT cells (Fig. 8), we checked the genes related to apoptosis (such as cleaved caspase-3, ATM, p53, ERK, JNK, and p38) by Western blot analysis. Radiation treatment increased the expression of ATM, P-p53 (serine 15), JNK, p38 and cleaved caspase-3. Co-treatment with KRG inhibited their expression.

## DISCUSSION

The aim of the present study was to examine the protective effect of KRG on irradiated normal human keratinocytes and to investigate possible mechanisms of protection. We demonstrated for the first time that treatment with KRG effectively protected HaCaT human keratinocytes from radiation, and we analyzed the intracellular apoptotic signal transduction pathway linked to the activation of caspase. Interestingly, we showed a different pattern of protective effects with the MTT and the colony-forming assays. In the MTT assay, KRG demonstrated a protective effect from 10 μg/ml, but this effect did not increase dose dependently; however, in the clonogenic assay, only the higher dose of 50 μg/ml of the compound produced such an effect. These results suggest that the delayed protective effect of KRG occurs at a higher concentration than the immediate protective effect against irradiation.

A large number of compounds that were initially promising to be radioprotective agents *in vitro* failed to reach the preclinical stage because of safety concerns *in vivo*. On the other hand, KRG is a dietary ingredient and has been used by humans for a long time, so the human safety of KRG has been proven [11, 18, 20, 21]. Furthermore, to test the safety of compounds and to investigate their protective effects in an *in vivo* system, we used the zebrafish model in our present and previous studies. The zebrafish, a small vertebrate species, has been widely used as an *in vivo* model system to study human disease because of its marked similarity to mammals in many of the key genes involved in developmental processes, cell cycle progression and proliferation, and differentiation [22]. Unlike other vertebrate species, however, zebrafish are rapidly bred and easily maintained in the laboratory. Zebrafish embryos provide a versatile model system for assaying radioprotectors/radiomitigators in a vertebrate organism, both on a systemic and organ-specific basis [23].

Although ionizing radiation-induced oral mucositis is a complicated process that involves the effects of irradiation on the keratinocytes and other cell populations in the submucosal tissue, such as fibroblasts and endothelial cells, monolayer cultures of keratinocytes are often used to assess the effects of ionizing radiation [24–26]. Tobita *et al*. recently developed an *in vitro* model for oral mucositis using HaCaT human keratinocytes [25]. Therefore, we used these cells to investigate radiation induction in an oral mucositis model.

ROS are generated in many stressful conditions and induce cell death by apoptosis or necrosis. We previously reported that cisplatin-induced and radiation-induced cellular toxicity is caused by ROS generation as well as changes in the MMP [9, 27]. In this study, we demonstrated radiation-induced apoptosis of HaCaT by an increase in TUNEL-positive cells, and that MMP and KRG prevented radiation-induced apoptosis of HaCaT in a dose-dependent manner by reduction of ROS and by stabilizing the change in MMP.

Increased intracellular ROS levels can activate ATM and its down-stream effector-p53 [28]. ATM is a key regulatory kinase activated by stress cues, which phosphorylates p53 on Ser^15^ and Ser^46^, thereby inducing apoptosis [10]. We examined the activation state of the ATM–p53–apoptosis pathway in HaCaT cells with/without KRG treatment. Indeed, radiation induced ATM activation, and KRG reduced expression of the ATM, in a dose-dependent fashion in HaCaT cells.

Increase in intracellular ROS levels can also induce mitogen-activated protein kinase (MAPK) activation [29]. MAPKs constitute a family of serine/threonine kinases that include extracellular signal-regulated kinase (ERK), JNK, and p38 MAPK. MAPKs play crucial roles in the integration of signal transduction leading to various cell responses such as cell proliferation, differentiation, and apoptosis [30]. Chronic activation of p42/44 MAPK may lead to cellular growth arrest, eventually inducing apoptosis [31]. A recent study demonstrated that gefitinib-induced apoptosis was mediated by the p38-JNK pathway in HaCaT cells [32]. In this study, irradiation caused the increase in activation of the JNK and p38 MAPK pathways in HaCaT cells. KRG reduced the radiation-induced activation of the JNK and p38 pathways, and bestowed cell protection by preventing apoptosis.

In summary, radiation-induced cell death in human keratinocyte HaCaT cells, which are a representative model of the oral mucosal squamous epithelium [26], occurred via an increase in membrane damage and intracellular ROS. KRG showed a radioprotective effect by reducing the radiation-induced increase in the level of ROS, the change in MMP, and the activation of the ATM–p53 and JNK–p38 pathways. Therefore, KRG exerts a protective effect against radiation-induced oral mucosal epithelium injury. This effect was confirmed using a zebrafish embryotoxicity model. These findings suggest that KRG can potentially be used as a protective agent against radiation-induced oral mucositis, which is a common complication of radiotherapy for head and neck cancers.
